# Revitalizing Muscle Repair: Hyaluronan Preserves Mitochondrial Architecture and Promotes Myogenesis Under Pro-Inflammatory Conditions

**DOI:** 10.3390/biom16060913

**Published:** 2026-06-19

**Authors:** Fabio Ferrini, Giosuè Annibalini, Michela Battistelli, SeyedehMahboobeh Moosavi, Osman Riham, Fabiana Fanelli, Italo Capparucci, Piero Sestili, Elena Barbieri

**Affiliations:** Department of Biomolecular Sciences, University of Urbino Carlo Bo, 61029 Urbino, Italy; fabio.ferrini@uniurb.it (F.F.); s.moosavi@campus.uniurb.it (S.M.); r.osman@campus.uniurb.it (O.R.); f.fanelli3@campus.uniurb.it (F.F.); italo.caparucci@uniurb.it (I.C.); piero.sestili@uniurb.it (P.S.); elena.barbieri@uniurb.it (E.B.)

**Keywords:** hyaluronic acid, myogenic differentiation, C2C12, inflammation, autophagy

## Abstract

Hyaluronic acid (HA), a major component of the glycome and a non-sulfated glycosaminoglycan, plays a crucial role in regulating stem cell behavior and function, thereby supporting skeletal muscle repair under inflammatory conditions. In this study, we investigated the effects of a mixture of HA fractions with different molecular weights (M-HA; 2–1000 kDa) on the repair capacity and myogenic potential of C2C12 murine myoblasts exposed to inflammatory stimuli. C2C12 cells were cultured, induced to differentiate, and treated with M-HA (1 mg/mL) under either physiological or inflammatory conditions (LPS, 10 µg/mL; IL-1β, 20 ng/mL). M-HA exhibited no cytotoxic effects, even at the highest concentration tested (1.0 mg/mL), and significantly enhanced scratch wound closure. Moreover, M-HA improved the myogenic index at day 5 of differentiation, promoted the expression of myogenic markers, preserved myosin heavy chain (MHC) levels under inflammatory stress, and reduced the expression of autophagy-related genes. Ultrastructural analyses revealed that untreated myotubes displayed swollen mitochondria, disrupted cristae architecture, and numerous autophagic vacuoles, whereas M-HA-treated cells exhibited well-preserved mitochondrial morphology, intact cristae organization, reduced cytoplasmic damage, and maintained myofibrillar structure. Taken together, the functional, molecular, and ultrastructural findings demonstrate that M-HA protects myoblasts from inflammation-induced cellular damage and supports their regenerative capacity. These results underscore the potential of glycomics-based strategies to enhance myogenic differentiation and promote skeletal muscle regeneration in inflammatory microenvironments.

## 1. Introduction

Degenerative diseases, surgical procedures, and traumatic injuries can lead to skeletal muscle damage, representing a major clinical challenge in sports medicine and orthopedics [1]. Despite the availability of several therapeutic approaches, including physiotherapy, pharmacological treatments, and surgical repair, complete restoration of muscle structure and function is often not achieved [2]. In this context, regenerative medicine has emerged as a promising strategy to enhance tissue repair and functional recovery. By combining biomaterials and bioactive molecules, regenerative approaches can promote cellular regeneration and tissue remodeling processes [3].

Among the biomaterials currently under investigation, hyaluronic acid (HA) has attracted considerable attention owing to its excellent biocompatibility and diverse biological functions. HA is a key component of the glycome, a non-sulfated glycosaminoglycan, and a major constituent of the extracellular matrix (ECM) [4]. Structurally, it consists of repeating disaccharide units of N-acetylglucosamine and glucuronic acid. Through its interaction with CD44, a transmembrane receptor expressed on the cell surface, HA regulates fundamental cellular processes including migration, proliferation, and inflammatory responses [5,6]. Owing to its unique mechanical and biochemical properties, HA has been increasingly explored as a therapeutic agent in regenerative medicine and muscle repair applications [7].

Recent evidence suggests that HA plays an important role in skeletal muscle regeneration by influencing muscle stem cell (MuSC) activation, proliferation, and differentiation. In particular, the expression of hyaluronan synthase 2 (HAS2) is regulated by the histone demethylase JMJD3, leading to increased HA production. The resulting HA-rich microenvironment promotes MuSC activation, modulates inflammatory responses, and supports muscle repair, highlighting the relevance of HA in the regeneration of injured skeletal muscle [8].

Importantly, the biological activity of HA is strongly influenced by its molecular weight [5]. High-molecular-weight HA (HMW-HA) generally exerts anti-inflammatory and cytoprotective effects, whereas low-molecular-weight HA (LMW-HA) is often associated with pro-inflammatory signaling. Consequently, HA plays a complex and dynamic role in regulating inflammation, extracellular matrix remodeling, and the structural organization of regenerating muscle tissue through molecular weight-dependent mechanisms [9].

Inflammation is a critical determinant of skeletal muscle repair and, when excessive or prolonged, can impair regenerative processes. Pro-inflammatory cytokines such as TNF-α and IL-1β negatively affect myogenesis and contribute to muscle atrophy. To mimic inflammatory conditions in vitro, lipopolysaccharide (LPS) and IL-1β are commonly employed as inflammatory stimuli. LPS reproduces bacterial-derived signals associated with post-traumatic infections and activates the NF-κB pathway in myoblasts through toll-like receptor 4 (TLR4). This activation reduces myoblast proliferation and impairs myogenic differentiation in developing myotubes. Similarly, IL-1β, a cytokine released during both acute and chronic muscle injury, suppresses myogenic differentiation by activating NF-κB signaling, inhibiting MyoD activity, and disrupting myogenic transcriptional programs, ultimately promoting muscle wasting [10,11,12,13]. Persistent inflammatory signaling may also activate the NLRP3 inflammasome, further compromising post-injury muscle healing [14].

In this context, HA may contribute to tissue repair by modulating the production of inflammatory cytokines, growth factors, and other regenerative mediators, thereby creating a microenvironment favorable for healing [15]. Skeletal muscle differentiation is orchestrated by a family of myogenic regulatory factors (MRFs), including MyoD, Myf5, myogenin, and MRF4. MyoD and Myf5 act during the early stages of myogenesis, regulating myoblast commitment and satellite cell proliferation, whereas myogenin and MRF4 are primarily involved in the later phases of differentiation and myofiber maturation [16]. Because inflammatory stress can disrupt this tightly regulated network, therapeutic strategies capable of preserving myogenic signaling under adverse conditions are highly desirable.

Beyond its direct effects on myogenic cells, HA also influences the activity and bioavailability of growth factors involved in muscle regeneration, including insulin-like growth factor-1 (IGF-1) and transforming growth factor-β (TGF-β). HA-based biomaterials functionalized with IGF-1-derived peptides have been developed to enhance myogenesis, promote neovascularization, reduce fibrosis, and stimulate regenerative signaling through activation of the IGF-1/AKT pathway [17]. These findings further support the use of HA-based biomaterials in skeletal muscle regenerative medicine. A notable example is represented by HA-based hydrogels, which provide a three-dimensional microenvironment conducive to muscle cell growth and differentiation [18,19]. Moreover, these systems can facilitate wound healing through the controlled release of regenerative factors and bioactive molecules [20]. Collectively, these studies highlight the versatility of HA as a platform for skeletal muscle tissue engineering.

Additional evidence has emerged from a specific HA formulation consisting of a mixture of low-, medium-, and high-molecular-weight HA fractions, designed to combine the complementary biological activities associated with each molecular weight range [21]. Such a formulation represents a promising candidate for skeletal muscle regeneration because of its potential to simultaneously attenuate inflammation and oxidative stress while supporting cellular differentiation and tissue repair. Nevertheless, limited information is currently available regarding its effects on skeletal muscle cells at the ultrastructural level, particularly under inflammatory stress conditions.

Therefore, the aim of the present study was to investigate the effects of a mixture of HA fractions with different molecular weights (M-HA; 2–1000 kDa, representative of commercially available preparations) on C2C12 murine myoblasts. Specifically, we evaluated cell viability, migration, and myogenic differentiation under inflammatory stress, with particular emphasis on functional outcomes, molecular markers of myogenesis, autophagy-related pathways, and ultrastructural alterations. Overall, this study provides new insights into the regenerative potential of M-HA and supports its use as a promising biomaterial for skeletal muscle repair.

## 2. Materials and Methods

### 2.1. Materials

M-HA, a blend with molecular weights ranging from 2 to 1000 kD (2/100/200/500/1000 kDa, Regenyal Laboratories Srl, San Benedetto del Tronto AP, Italy) was used in this study. The formulation consisted of extensively purified, pharmaceutical-grade HA obtained by fermentation of *Streptococcus equi* (e.g., purity > 95%, water content < 10%, EU/mg < 0.05, and very low metal content).




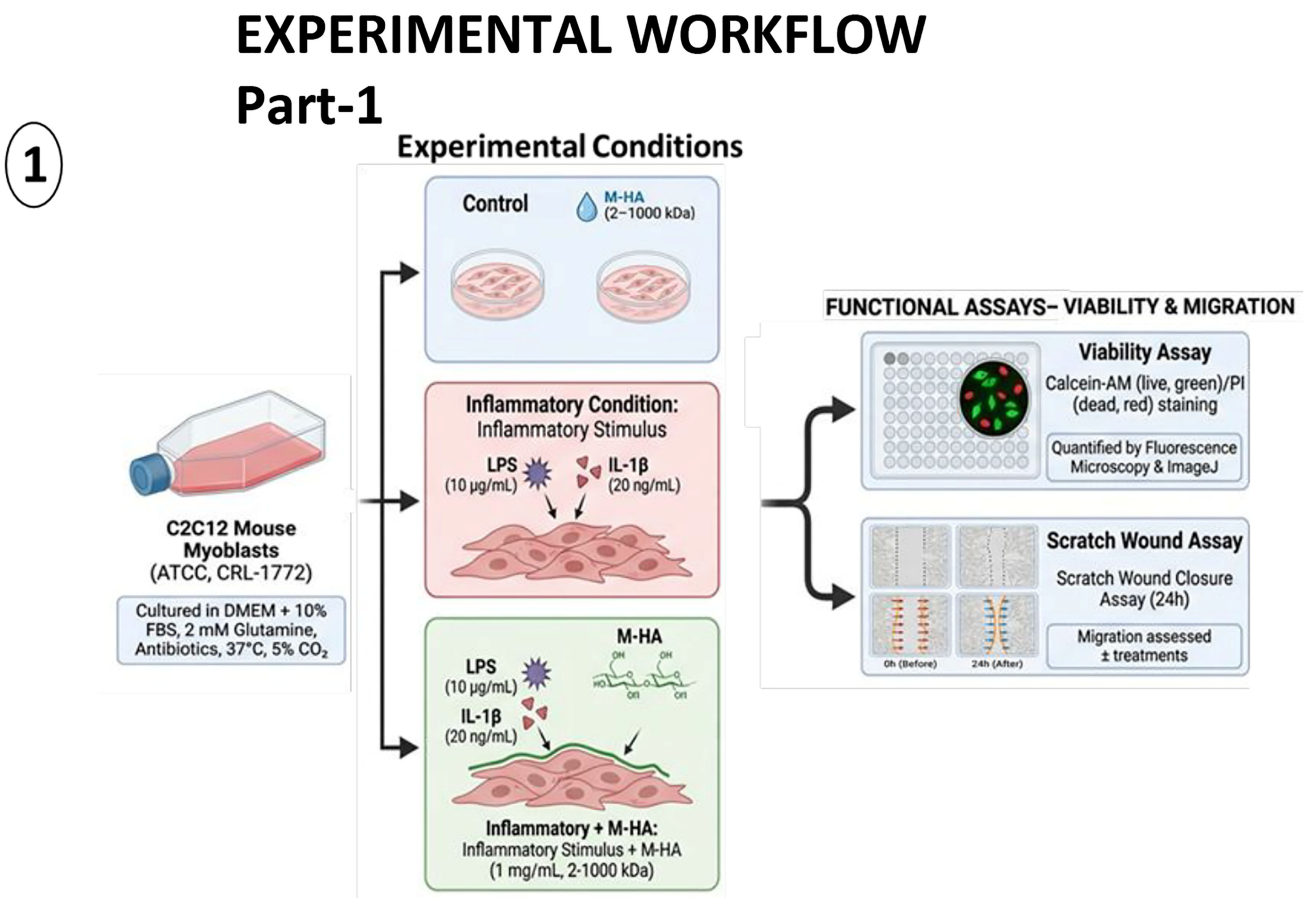



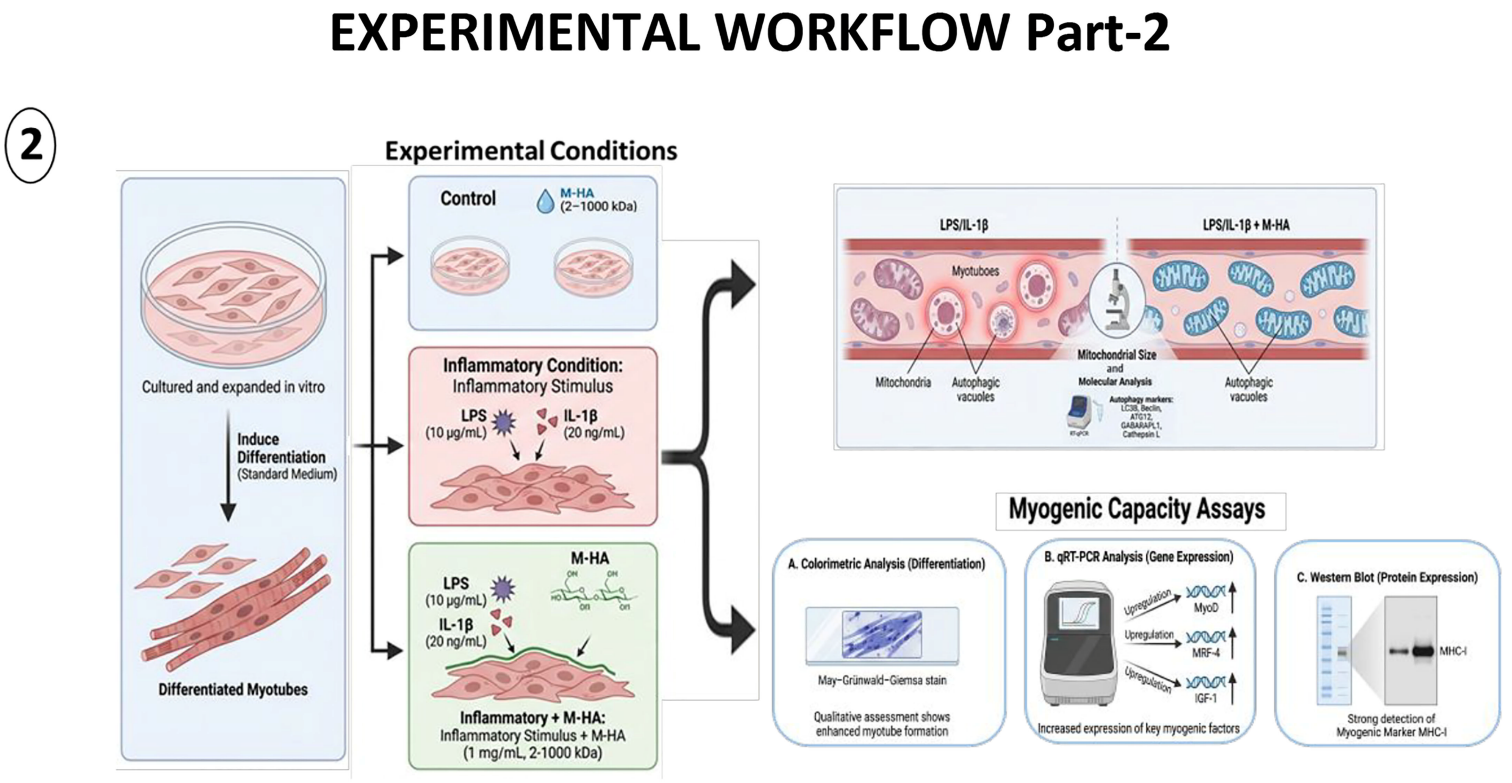




### 2.2. Cell Culture and Treatment Conditions

C2C12 myoblast cells were divided into four experimental groups: Control group (CNT): untreated cells (no intervention); M-HA group: cells treated with M-HA (1 mg/mL); Stress group: cells exposed to inflammatory stimuli (LPS 10 μg/mL and IL-1β 20 ng/mL); and Stress with M-HA group: cells treated simultaneously with M-HA and inflammatory stimuli. M-HA was added during the first 24 h of differentiation (T0–T1), while inflammatory stimuli (stress) were applied from T1 to T5. The rate of cell proliferation and differentiation was assessed at designated time points, and specific assays were performed as described below.

C2C12 mouse myoblasts (CRL-1772 ATCC) were cultured in Dulbecco’s Modified Eagle Medium (DMEM) (Sigma-Aldrich, Milan, Italy) supplemented with 10% *v*/*v* fetal bovine serum (Sigma-Aldrich, Italy), 2 mM glutamine (Sigma-Aldrich, Italy), and antibiotics (50 U/mL penicillin, 50 μg/mL streptomycin, Sigma-Aldrich, Italy) for 24–48 h to reach 80% of cell confluence. C2C12 myoblast differentiation was induced by serum withdrawal 1% *v*/*v* (differentiating medium (DM)). Myogenic differentiation was performed as described in [22]. Cells were observed and processed at critical time points, i.e., selected differentiation days (T), referred to as “Tn,” where “n” represents the number of days from the commencement of differentiation.

M-HA loading of C2C12 was carried out by adding 1 mg/mL to myoblasts during the first 24 h of differentiation. At this time point (i.e., at the end of day 1, T1), M-HA-supplemented cultures were exposed to LPS 10 µg/mL, IL-1ꞵ 20 ng/mL in DM up to day 5 (T5).

In order to assess the pro-proliferative activity of M-HA, the monolayer of myoblasts grew in complete DMEM from T0 to T1 (after 24 h) in the presence/absence of 1 mg/mL M-HA and with/without pro-inflammatory agents such as the lipopolysaccharide (LPS) 10 µg/mL and the interleukin (IL)-1ꞵ 20 ng/mL, slowing down their proliferation.

THP-1 cells (ATCC, Manassas, VA, USA) were routinely grown in RPMI 1640 supplemented with 10% heat-inactivated FBS and antibiotics (100 units/mL penicillin, 100 µg/mL streptomycin) and 2 mM L-glutamine. Cells were maintained in a humidified incubator at 37 °C with 5% CO_2_. 

### 2.3. Cell Viability

We performed a calcein-AM/propidium iodide staining assay to evaluate the cell viability based on the following steps. Briefly, at the first step, C2C12 myoblasts were 5 × 10^4^ cells/cm^2^ seeded in 6-well tissue culture (TC) plates, treated with 1 mg/mL of M-HA, and grown in complete DMEM for up to 48 h. After incubation, at the second step, cells were washed with phosphate-buffered saline (PBS) and then incubated with a staining solution containing 2 µM calcein-AM and 4 µM propidium iodide (Thermo Fisher Scientific, Waltham, MA, USA) in PBS for 20 min at 37 °C in the dark. Calcein-AM is a non-fluorescent dye that can only enter live cells and be hydrolyzed by intracellular esterases to produce green fluorescent signals (excitation/emission: 495/515 nm). However, PI penetrates only cells with damaged membranes and binds to nucleic acids to produce red fluorescent signals (excitation/emission: 535/617 nm). Next, this fluorescence was observed and acquired at 10× magnification using an inverted fluorescent microscope (Olympus IX51, Olympus Corporation, Tokyo, Japan). Finally, the ratio of live cells to dead cells, which were calcein-positive and PI-negative, respectively, was quantified using ImageJ software and showed as a percentage of viable cells relative to the total number of stained cells.

### 2.4. Scratch Repair Test

We performed the scratch repair assay to assess the migration capacity. In this assay, first, 5 × 10^4^ cells/cm^2^ C2C12 cells were seeded in a six-well TC plate and incubated at 37 °C and 5% CO_2_ to grow and create a monolayer. Second, a scratch wound was created horizontally along the flask, using the tip of a sterilized pipette. Third, the medium of each well was removed gently and the cells were washed with PBS solution and then grown for 24 h with fresh medium. It should be noted that this medium was used with and without 1 mg/mL of M-HA and inflammatory stimuli (LPS 10 µg/mL, IL-1ꞵ 20 ng/mL). The untreated cells were used as a control. Fourth, pictures of the scratch were taken both immediately after scratching (t0) and after 24 h (t1) using a phase contrast microscope (Olympus IX51 4× objective). Subsequently, the images were analyzed to evaluate the wound closure using the ImageJ software 1.52a. Finally, the extent of wound closure was calculated as follows: (Distance of original wound gap − Distance of remaining wound gap)/Distance of original wound gap × 100% [23].

### 2.5. Estimation of Myogenic Index

The myogenic index quantifies myogenic differentiation: the proportion of myotube nuclei among the total number of myonuclei. May–Grunwald–Giemsa-stained nuclei were observed at T5. The myogenic index was determined in five fields in a 60 mm dish for each kind of treatment in the microscope, and the number of cells was calculated at 300–500 nuclei per area [24].

For May–Grünwald–Giemsa staining, May–Grünwald solution was diluted 1:3 in sodium phosphate buffer (pH = 6) and incubated on the cells for 5 min. The cells were then washed with distilled water and placed in Giemsa solution at a dilution of 1:10 for 10 min. Finally, after washing twice with distilled water, the cells were observed and imaged using an inverted microscope [10].

### 2.6. Ultrastructural Analysis by Transmission Electron Microscopy

Ultrastructural analysis by transmission electron microscopy (TEM) was carried out to assess mature myotube morphology at T5 in detail and to identify potential cytoprotective effects of M-HA against stress- and inflammatory-induced insults. Particular attention was given to mitochondrial swelling, cristae organization, and autophagic vacuoles. For TEM observations, the C2C12 myotubes were treated as described in Garcia-Merino et al. 2024 [24]. C2C12 myotubes were washed and immediately fixed with 2.5% glutaraldehyde in 0.1 M phosphate buffer (pH 7.3) for 30 min at room temperature, then gently scraped and after another 30 min of fixation centrifuged at 1200 rpm. Pellets were additionally fixed for 1 h in 1% OsO4 for 1 h, alcohol dehydrated, and embedded in araldite. Thin sections were stained with UranyLess and lead citrate and analyzed using a Philips CM10 transmission electron microscope (FEI Italia SRL, Milano, Italy) as described in D’Emilio et al. 2010 [25]. Mitochondrial morphology, cristae organization, and autophagic vacuoles were evaluated. We also evaluated the myofibrilles presence in differentiated cells.

### 2.7. Quantitative Reverse Transcription PCR

The total RNA from the treated myotubes (T5) was extracted according to the E.Z.N.A. total RNA kit manual (VWR International, Milan, Italy). An E.Z.N.A RNase-Free DNase I set (VWR International, Milan, Italy) digestion step was performed on all of the RNA samples before subsequent reactions. The RNA concentration was estimated spectrophotometrically at 260 nm (SpectraMax QuickDrop Micro-Volume Spectrophotometer, Molecular Devices, San Jose, CA, USA). Reverse transcription of cDNA was performed from 500 ng of total RNA using the PrimeScript™ RT Reagent Kit (Takara Bio Europe, Saint-Germain-en-Laye, France). From the mRNA expression analyses, the cDNA products were subjected to real-time SYBR-green-based quantitative PCR on a StepOnePlus™ real-time PCR system (Applied Biosystems, Monza, Italy) in a final volume of 20 μL using a Power Up master mix (Life Technologies, Carlsbad, CA, USA) and 0.3 μM of the primer pairs reported in Table 1.

The primers used for the PCR experiments were previously designed and used by Sestili et al. and Annibalini et al. [16,26].

The relative levels of target mRNA expression were normalized to values obtained for the glyceraldehyde-3-phosphate dehydrogenase (GAPDH), used as a “housekeeping gene” simultaneously with the experimental samples. The RT–PCR conditions were as follows: 50 °C for 2 min, 95 °C for 2 min, followed by 40 cycles of two steps—95 °C for 15 s and 60 °C for 60 s—and, finally, three steps—95 °C for 15 s, 60 °C for 60 s, and 95 °C for 15 s. The product specificity was examined using dissociation curve analysis. The results were calculated using the ΔΔCt (2^−ΔΔCt^) method and expressed as a relative change compared with the untreated control (CNT), where the control is set to 1. Each sample was tested in triplicate by RT–qPCR.

### 2.8. Western Blot Analysis

For electrophoresis, the samples—after being collected at the T5 differentiation time point for each condition—were loaded onto a 12% SDS-PAGE gel.

Subsequently, proteins were blotted to a nitrocellulose membrane (GE Healthcare, Milan, Italy). The primary antibodies used were against Anti-Fast Myosin skeletal heavy chain (MHC, 1:1000 dilution, clone ab91506, Abcam, Cambridge, UK). Primary antibodies were incubated overnight at 4 °C, followed by washing and the application of a secondary HRP-conjugated antibody (Pierce, Thermo Fisher). Immune complexes were visualized using the Supersignal Dura reagent (Pierce, Thermo Fisher), and the autoradiographic films were quantified using ImageJ software v. 1.8.0 [27].

### 2.9. Statistical Analysis

Unless noted otherwise, the results were expressed as mean values ± SD. The statistical analyses were performed using GraphPad Prism version 8.02 for Windows. In all experimental sets, multiple comparisons were carried out between different pairs of groups using two-way ANOVA with Sidak’s multiple comparison test. Results were considered significant at a significance level of *p* < 0.05. All experiments were conducted in triplicate.

## 3. Results

### 3.1. Cell Viability

**Figure 1:**
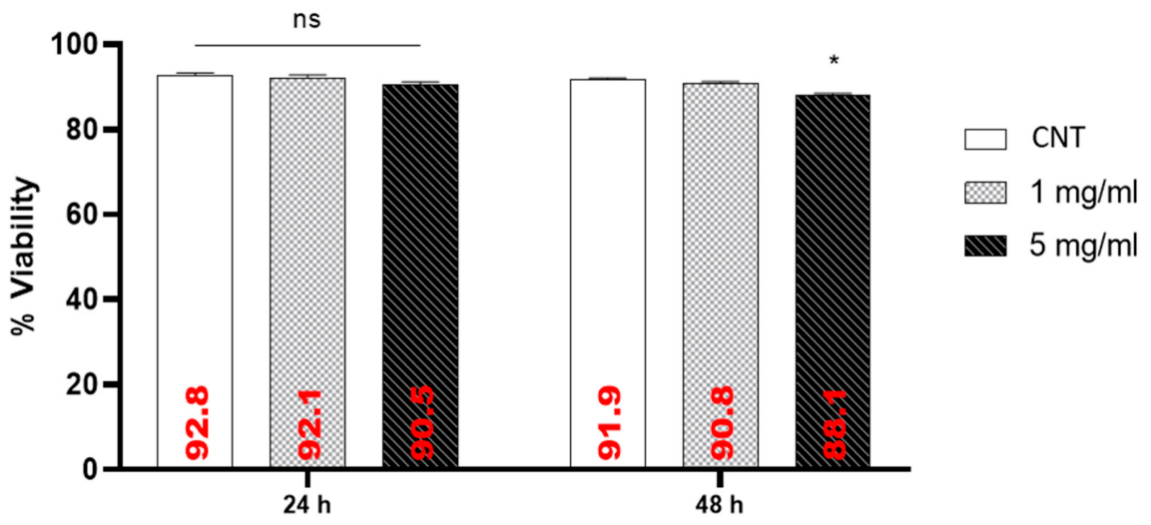
Viability of C2C12 myoblasts treated with 1 or 5 mg/mL concentrations of M-HA for 24 and 48 h, as assessed by calcein-AM/propidium iodide. Data are expressed as mean ± SD from *n* = 3 independent experiments, each performed in triplicate. Statistical analysis was carried out by two-way ANOVA—Sidak’s multiple comparisons test; * *p* < 0.05, CNT vs. M-HA, ns = not significant.

The calcein-AM/propidium iodide staining assay was used to assess the cell viability of M-HA on C2C12 myoblast viability. Figure 1 shows that M-HA did not affect myoblasts’ viability up to 48 h incubation, except for at 5.0 mg/mL, which caused a slight reduction at 48 h. These data confirm that concentrations of M-HA ranging from 1 to 5 mg/mL do not exert toxic effects under the experimental conditions tested. Trypan Blue exclusion assays showed that THP-1 cell viability was largely preserved after treatment with M-HA at 1 and 5 mg/mL for 24 and 48 h (Supplementary Materials Figure S1).

### 3.2. Scratch Wound Repair

**Figure 2:**
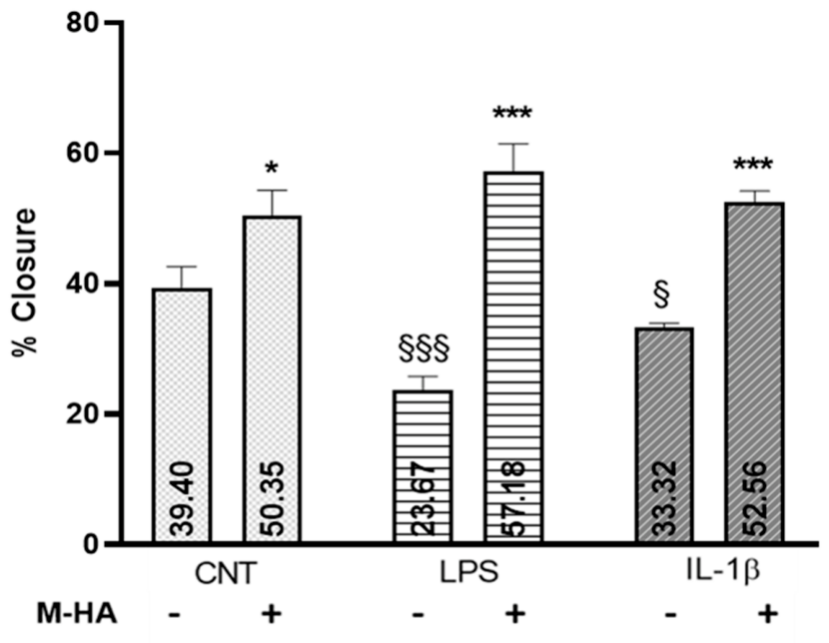
Effect of M-HA exposure on C2C12 myoblast migration under inflammatory conditions. The percentage of wound closure was evaluated 24 h after mechanical stress. Closure was assessed by incubating cells with 10 µg/mL LPS and 20 ng/mL IL-1β in the absence or presence of M-HA (1 mg/mL). Data are expressed as mean ± SD from *n* = 3 independent experiments, each performed in triplicate. Statistical analysis was carried out by two-way ANOVA–Sidak’s multiple comparisons test; * *p* < 0.05, *** *p* < 0.005, Stress vs. Stress + M-HA. Control vs. Stress § *p* < 0.05, §§§ *p* < 0.001.

C2C12 myoblast confluent monolayers were mechanically scratch-wounded with a sterile tip. Results depicted in Figure 2 show that M-HA (1 mg/mL) significantly increased the scratch closure capacity of C2C12 myoblasts (39.40% vs. 50.35%, *p* < 0.05, CNT vs. M-HA 1 mg/mL).

The wound closure was significantly impaired when cultures were incubated in the presence of 10 µg/mL LPS and 20 ng/mL IL-1β, both included as representative pro-inflammatory stimuli (23.67, 33.32% vs. 39.40%, *p* < 0.001, *p* < 0.05, respectively, LPS and IL-1β vs. CNT). Interestingly, the addition of M-HA prevented LPS and IL-1β wound closure impairment ([LPS 23.67% vs. 57.18%, *p* < 0.005]; [IL-1β 33.32% vs. 52.56%, *p* < 0.005], Figure 2).

### 3.3. Myogenic Differentiation

**Figure 3:**
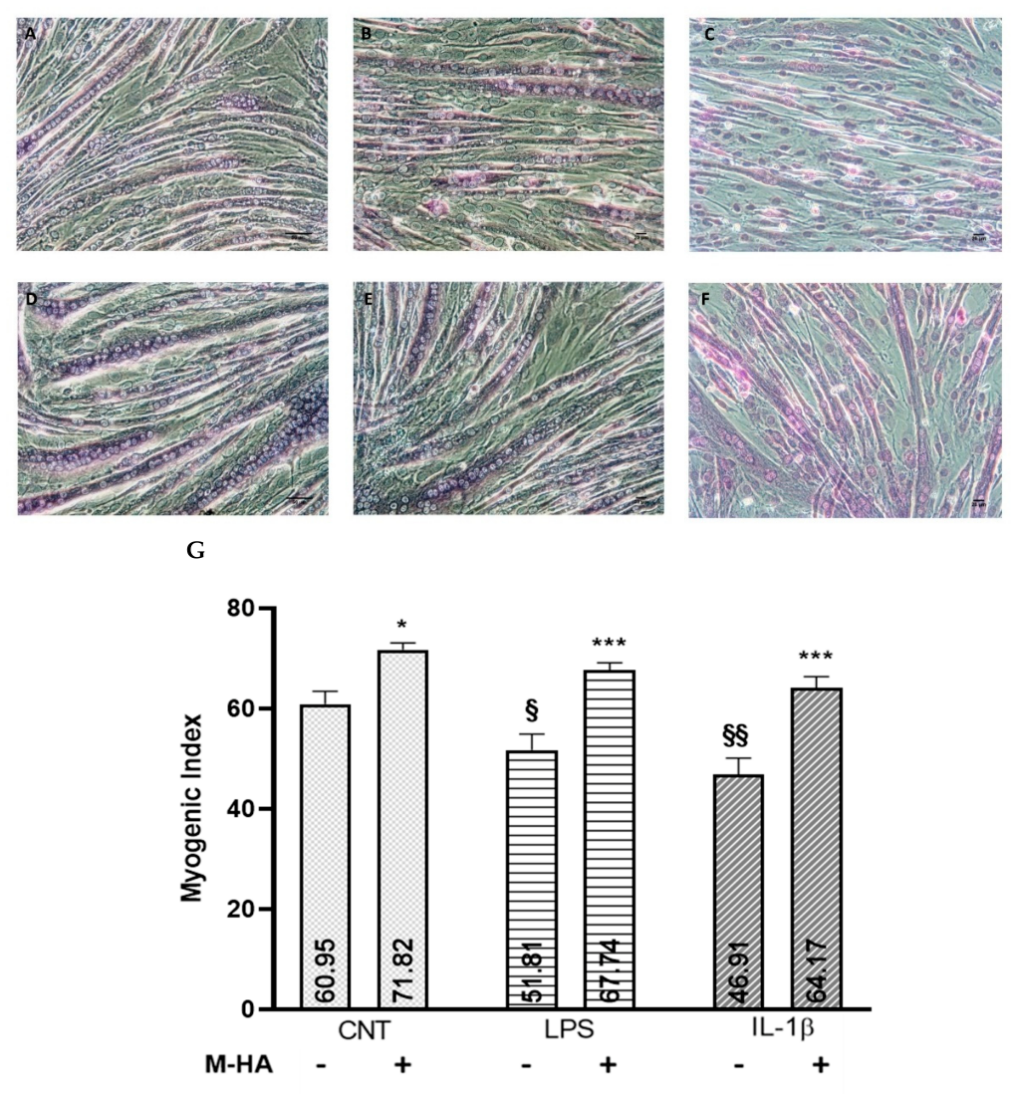
Effect of M-HA on C2C12 myogenic index under control or experimental conditions. (**A**) Representative images of C2C12 myotubes stained with May–Grünwald–Giemsa (CNT); (**D**) cells treated with 1.0 mg/mL M-HA. C2C12 were exposed to pro-inflammatory stress using LPS or IL-1β in absence (−) (**B**,**C**) or presence (+) (**E**,**F**) of M-HA. (**G**) Quantitative analysis of the myogenic index. Five random fields/60 mm dish (*n*  =  3 independent experiments) were observed; the total number of nuclei analyzed was 300–500/field. Statistical analysis was carried out by two-way ANOVA; * *p* < 0.05, Control vs. M-HA and *** *p* < 0.005 Stress vs. Stress + M-HA; Control vs. Stress § *p* < 0.05, §§ *p* < 0.005.

Myogenic index quantification at day 5 (T5) revealed that the percentage of multinucleated myotubes, as well as the abundance of nuclei per myotube, were significantly higher in the M-HA-treated groups as compared with the untreated ones ([71.24 vs. 60.95, *p* < 0.05], Figure 3). Similarly to the effects on scratch closure capacity shown in Figure 2, the presence of LPS and IL-1β remarkably decreased the myogenic index of differentiating C2C12 cells ([51.81–46.91 vs. 60.95%, *p* < 0.05–*p* < 0.01], Figure 3). Interestingly this effect could be prevented by co-incubating cells with M-HA ([LPS—66.74 vs. 51.81 *p* < 0.005; IL-1β—64.17 vs. 46.91 *p* < 0.005], Figure 3).

### 3.4. Ultrastructure Analysis

**Figure 4:**
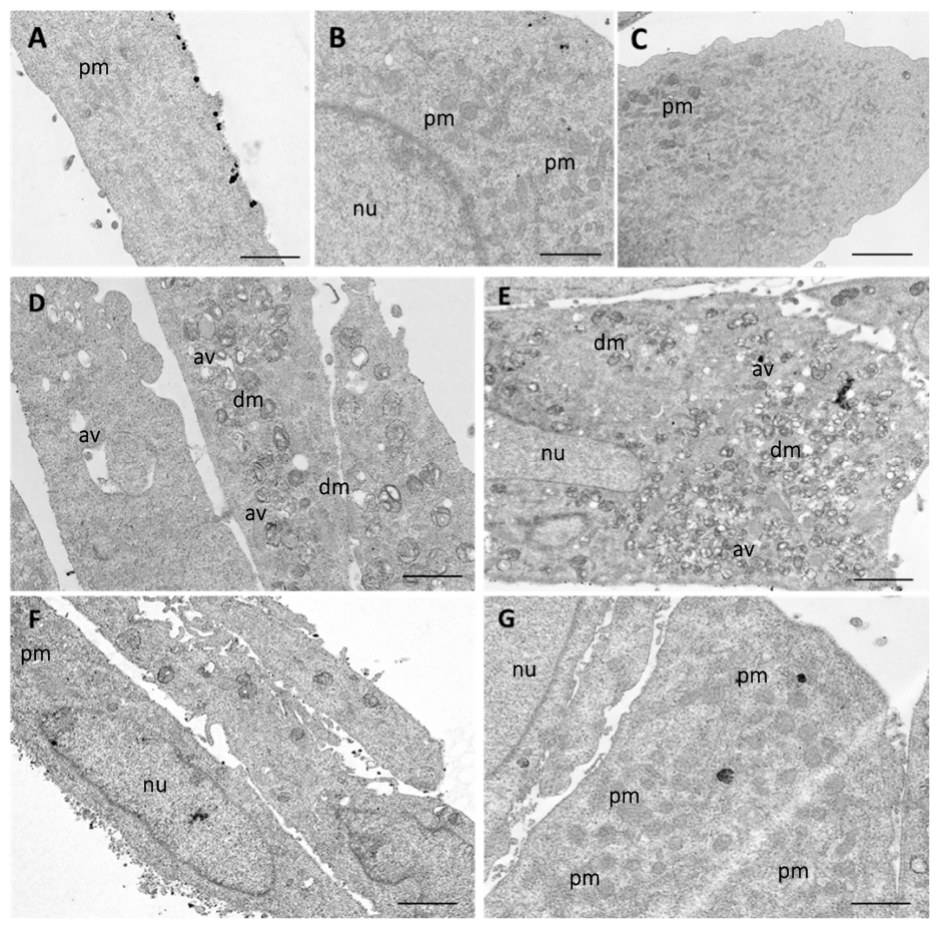
Transmission electron microscopy of C2C12 myoblasts shows preserved cell morphology in control cells (**A**) and those treated with 1.0 mg/mL M-HA (**B**,**C**), including intact nuclei and mitochondria. Cells treated with LPS (**D**), or IL-1β (**E**) show reduced cell size, mitochondria swelling, cristae disruption (dm), and accumulation of autophagic vacuoles (av). Co-treatment with M-HA markedly attenuates these changes in both LPS- (**F**) and IL-1β-treated cells (**G**), preserving nuclei (nu) and mitochondria (pm), particularly in IL-1β + M-HA conditions. Scale bars: (**A**,**C**,**D**,**F**) = 5 μm; (**B**) = 1 μm; (**E**,**G**) = 2 μm. nu = nuclei; pm = preserved mitochondria; dm = disrupted mitochondria; av = autophagic vacuoles.

The ultrastructure of treated and untreated differentiating myoblasts was evaluated by TEM (Figure 4). In both control and M-HA-treated C2C12, cells exhibited a well-defined cellular structure and morphology. Nuclear membranes appear well preserved, and diffuse chromatin could be visible inside the nuclei. Numerous conserved organelles and mitochondria surrounding the nuclei were observed. Mitochondria show a preserved double membrane and well-organized cristae. In contrast, the cells treated with LPS (Figure 4D) and IL-1β (Figure 4E) showed morphological alterations. The mitochondria frequently appear swollen, and many of them show disrupted or poorly defined cristae. In the cytoplasm, numerous small and large autophagic vacuoles appear, consistent with stress-associated cellular damage.

The treatment with M-HA produces a protective effect against both LPS (Figure 4F) and IL-1β (Figure 4G). Indeed, upon addition of M-HA, cells retained a conserved nucleus morphology, with structurally preserved nuclear membrane and diffuse chromatin; in the cytoplasm, autophagic vacuoles disappear, and there are numerous intact mitochondria similar to control cells. These ultrastructural findings indicate that M-HA has a protective role against stress-related mitochondrial damage and that it limits cytoplasmic degenerative features.

During the evaluation of cellular morphology, we focused our attention on cytoplasmic organelles, in particular on mitochondrial ultrastructure and dimensions, given their crucial role in muscle cell function. Mitochondria were evaluated in ten different cells for four different samples.

**Figure 5:**
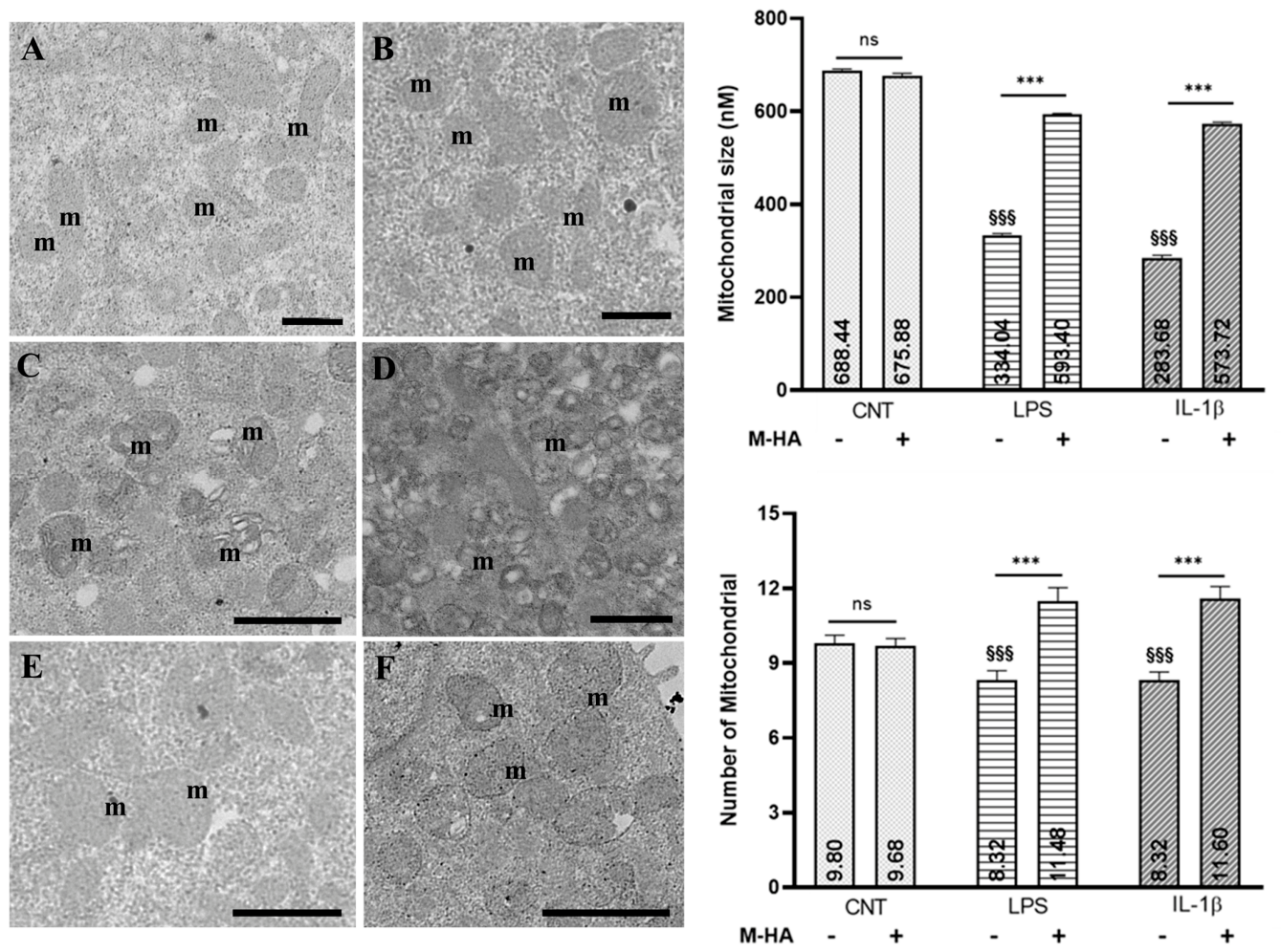
Mitochondrial ultrastructure in C2C12 cells under inflammatory conditions and after M-HA treatment. Control cells (**A**) and cells treated with M-HA, 1.0 mg/mL (**B**), show well-preserved mitochondria. Exposure to LPS (**C**) or IL-1β (**D**) caused mitochondrial alterations, including reduced size, membrane disruption, and disorganized cristae. Co-treatment with M-HA (**E**,**F**) markedly attenuates these changes, with mitochondria comparable to controls. Scale bar: (**A**–**F**) = 1 μm. m = mitochondria. *** *p* < 0.005, §§§ *p* < 0.001, ns = not significant.

**Figure 6:**
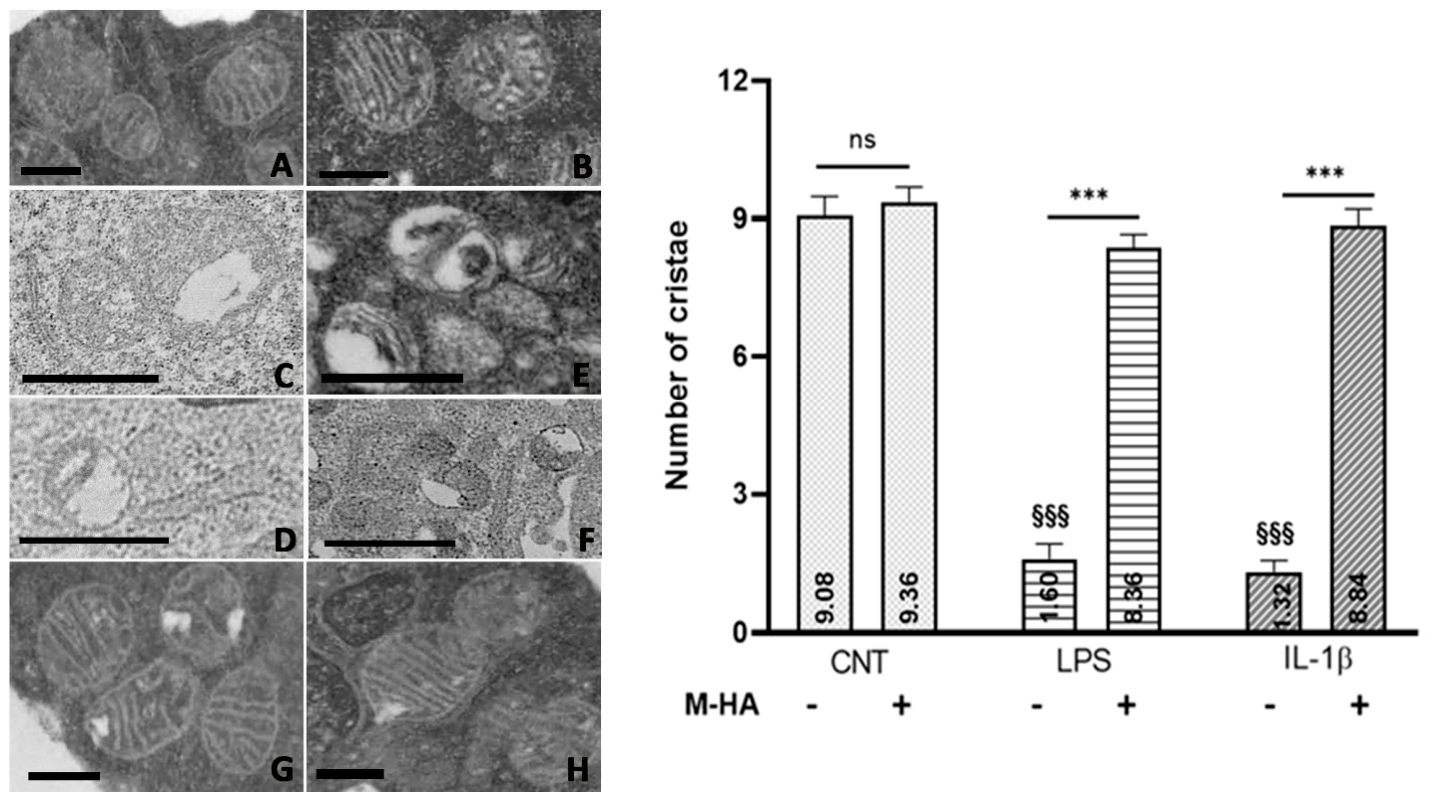
Ultrastructural analyses of C2C12 mitochondria in control and treated cells. Control cells (**A**) and those treated with 1.0 mg/mL M-HA (**B**) show well-preserved mitochondria with organized cristae. Exposure to LPS (**C**,**D**) or IL-1β (**E**,**F**) induced mitochondrial swelling, cristae disruption, reduced cell size, and mitophagy with RER closely opposed to mitochondria (**D**,**F**). Co-treatment with M-HA (**F**–**H**) markedly attenuates these changes, resulting in well-conserved mitochondria. Scale bars: (**A**–**H**) = 500 nm. *** *p* < 0.005, §§§ *p* < 0.001, ns = not significant.

Control and M-HA-treated cells showed well-preserved mitochondria, with average sizes of 690 and 670 nanometers, respectively (Figure 5A,B and Figure 6A,B). Under these conditions, about 10 mitochondria per 10 × 10 µm^2^ area were observed, displaying well-conserved cristae (Figure 5A,B and Figure 6A,B). In contrast, LPS- and IL-1β-treated cells showed mitochondrial damage. Several mitochondria had lost their cristae and appeared smaller, with average sizes of about 340 and 290 nm, respectively (Figure 5C,D and Figure 6C–F). Evidence of mitophagy was also observed under these conditions, as indicated by the presence of rough endoplasmic reticulum (RER) in proximity to mitochondria (Figure 6D,F). Accordingly, the number of mitochondria was also reduced after treatment (Figure 6 (right panel)).

M-HA attenuated the damaging effects of LPS and IL-1β, as suggested by the preservation of mitochondrial membrane and cristae (Figure 5E,F and Figure 6G,H). Furthermore, in these conditions, the mitochondrial sizes were approximately 590 nm and 570 nm, respectively, and the number of mitochondria increased compared with LPS- and IL-1β-treated cells (Figure 5 and Figure 6 (right panel)).

### 3.5. Autophagy Biomarker Expression

**Figure 7:**
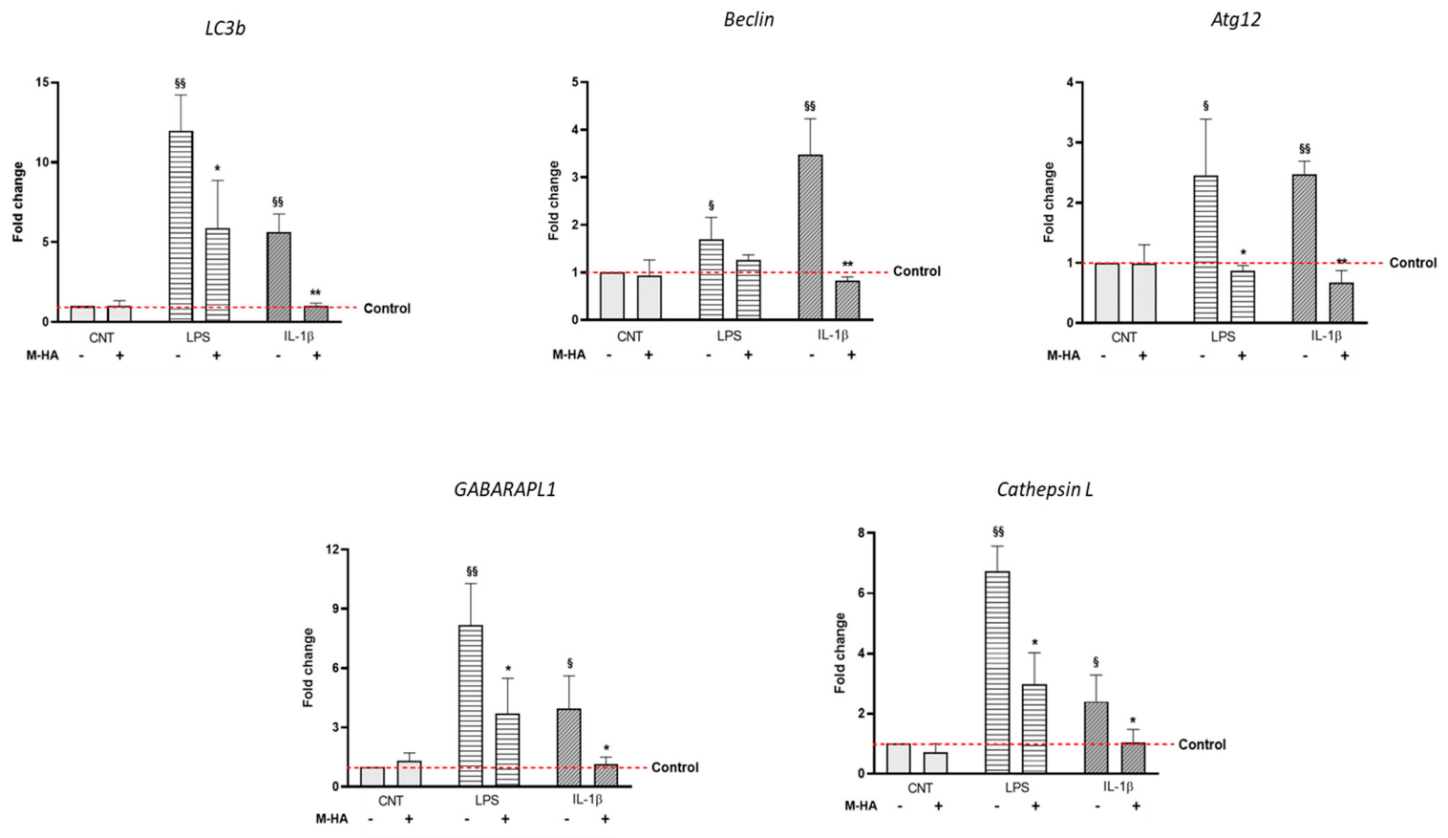
Gene expression of autophagy markers in myotubes. Myotubes were co-treated throughout the differentiation phase (from T0 to T5) with inflammatory stimuli and M-HA (1 mg/mL). Data are presented as mean ± SD from *n* = 3 independent experiments, each performed in triplicate. Statistical significance was determined by two-way ANOVA–Sidak’s multiple comparisons test (* *p* < 0.05; ** *p* < 0.01; Control vs. Stress § *p* < 0.05, §§ *p* < 0.001).

Data obtained through qRT–PCR indicate that inflammatory stimuli strongly upregulate certain autophagy-related genes in C2C12 differentiated myotubes (T5), under LPS and IL-1β exposure ([LC3-b fold changes, 11.95 and 5.62, respectively]; [beclin fold changes, 1.70 and 3.473, respectively]; [ATG12 fold changes, 2.457 and 2.476, respectively]; [GABARAPL1 fold changes, 8.168 and 3.954, respectively]; [cathepsin L fold changes, 6.725 and 2.409, respectively] Figure 7), indicating activation of an autophagy-associated transcriptional program. Notably, M-HA treatment markedly attenuated this response without affecting basal gene expression, suggesting a modulatory rather than inhibitory role (M-HA vs. LPS and IL-1β + M-HA [LC3-b fold changes, 5.865 and 1.010, respectively]; [beclin fold changes, 1.262 and 0.8225, respectively]; [Atg12 fold changes, 0.868 and 0.6712, respectively]; [GABARAPL1 fold changes, 3.694 and 1.130, respectively]; [cathepsin L fold changes, 2.984 and 1.040, respectively]). These results suggest that M-HA limits the activation of autophagy-associated pathways triggered by inflammatory cues, potentially through modulation of upstream inflammatory signaling, thereby contributing to the preservation of myoblast homeostasis under stress conditions.

### 3.6. Expression of Myogenic Markers

**Figure 8:**
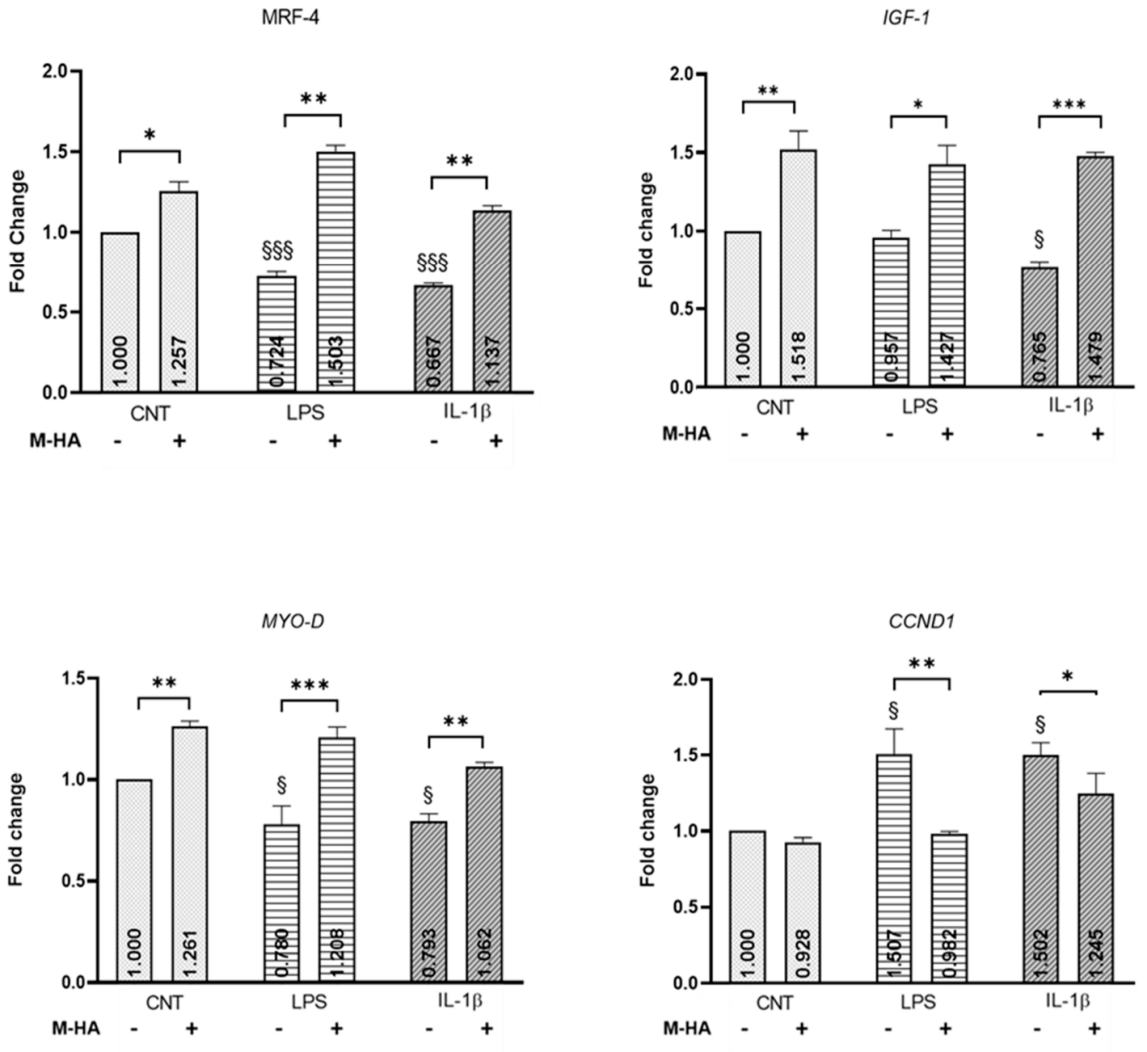
Gene expression of myogenic markers in C2C12 myoblasts. RT–qPCR analysis revealed that M-HA (1 mg/mL) upregulated the transcription of MyoD, MRF4, and IGF-1, while significantly downregulating CCND1, compared with untreated controls. C2C12 cells were treated throughout the differentiation phase (from T0 to T5) with inflammatory stimuli and M-HA (1 mg/mL). Data are presented as mean ± SD from *n* = 3 independent experiments, each performed in triplicate. Statistical significance was determined by two-way ANOVA–Sidak’s multiple comparisons test (* *p* < 0.05; ** *p* < 0.01; *** *p* < 0.005; Control vs. Stress § *p* < 0.05, §§§ *p* < 0.001).

The result of RT–qPCR analysis (Figure 8) showed that M-HA affected the transcriptional expression of myogenic regulatory factors (MRFs). In particular, M-HA increased the expression of MyoD ([fold change 1258, *p* < 0.01]) and MRF4 ([fold change 1257, *p* < 0.05]). IGF-1 expression was also increased by M-HA treatment ([fold change 1.499, *p* < 0.01]), a fact that might contribute to the establishment of a pro-differentiation environment.

Results in Figure 8 (downmost panels) indicate that the expression of CCND1, whose products counteract myogenesis, was not affected by the presence of M-HA.

We then evaluated the same panel of markers in differentiating inflammatory challenged myoblasts (LPS or IL-1β), in the presence or absence of M-HA. As expected, both triggers markedly affected the expression pattern of MRFs, with MyoD and IGF-1 significantly reduced. Notably, in these conditions, M-HA mitigated inflammation-associated dysregulation and partially restored the expression of these key pro-myogenic factors. In contrast, CCND1 exhibited a stimulus-dependent response. Overall, the meaning of these data is twofold: the above inflammatory-linked transcriptional changes disfavor the progression of C2C12 differentiation, while M-HA is capable of preventing this impairment.

**Figure 9:**
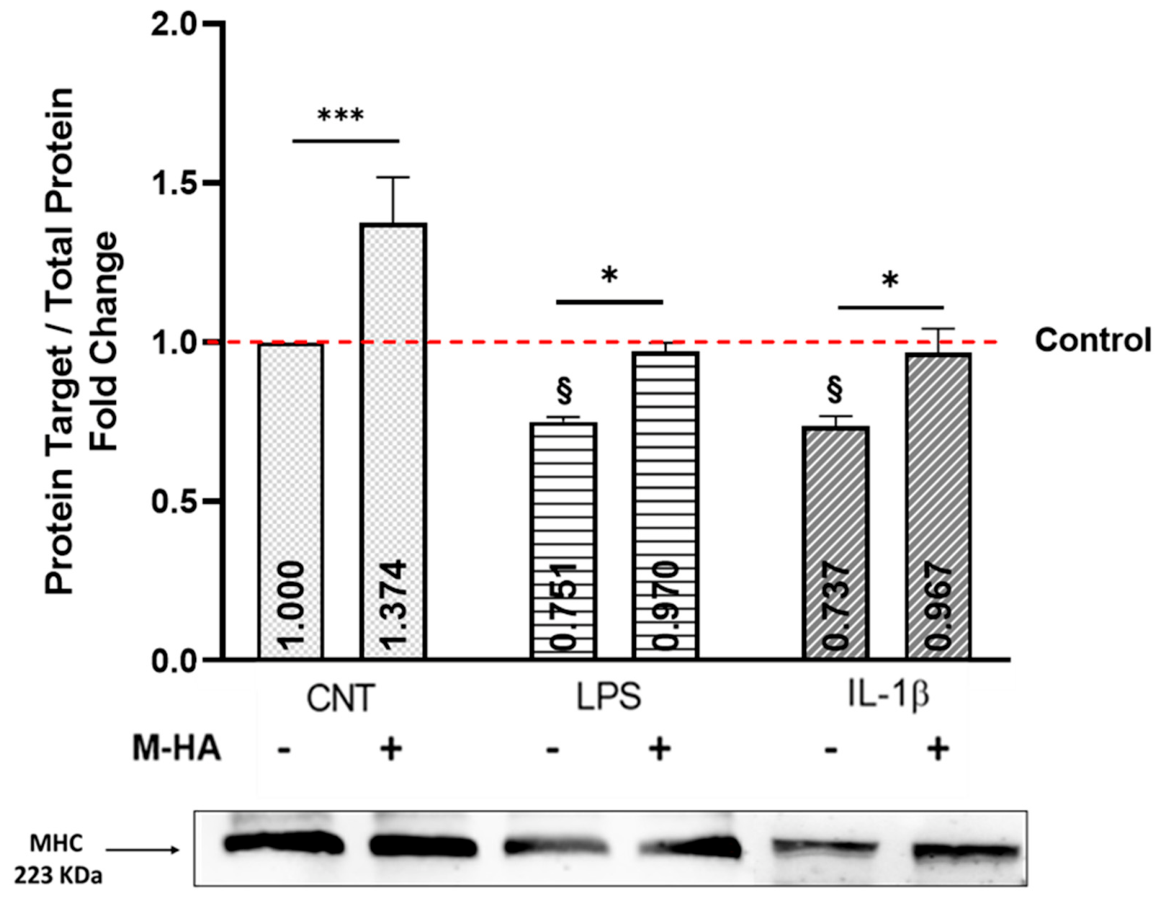
Myosin heavy chain (MHC) profile in myotube exposed to inflammatory stress during the differentiation phase (T5) with M-HA. Concentrations used: M-HA 1 mg/mL; IL-1B 20 ng/mL; LPS 10 µg/mL. Statistical analysis was carried out by two-way ANOVA; * *p* < 0.05, Control vs. M-HA and *** *p* < 0.001 Stress vs. Stress + M-HA; Control vs. Stress § *p* < 0.05 (see Figure S2 for the original image).

This inference is further strengthened by the observation that (Figure 9) IL-1β and, more markedly, LPS caused a decrease in MHC (CNT vs. LPS: 1.00 vs. 0.751; *p* < 0.05; CNT vs. IL-1β: 1.000 vs. 0.737, *p* < 0.05), indicating a loss of the differentiated myogenic phenotype and a shift toward a more proliferative state; the addition of M-HA partially attenuated these inflammation-linked effects, rescuing the execution of the myogenic program (MHC fold change: CNT vs. M-HA: 1.00 vs. 1.374, *p* < 0.05; IL-1β vs. IL-1β + M-HA: 0.7370 vs. 0.9674, *p* < 0.05; LPS vs. LPS + M-HA: 0.751 vs. 0.970, *p* < 0.05) (Figure 9).

**Figure 10:**
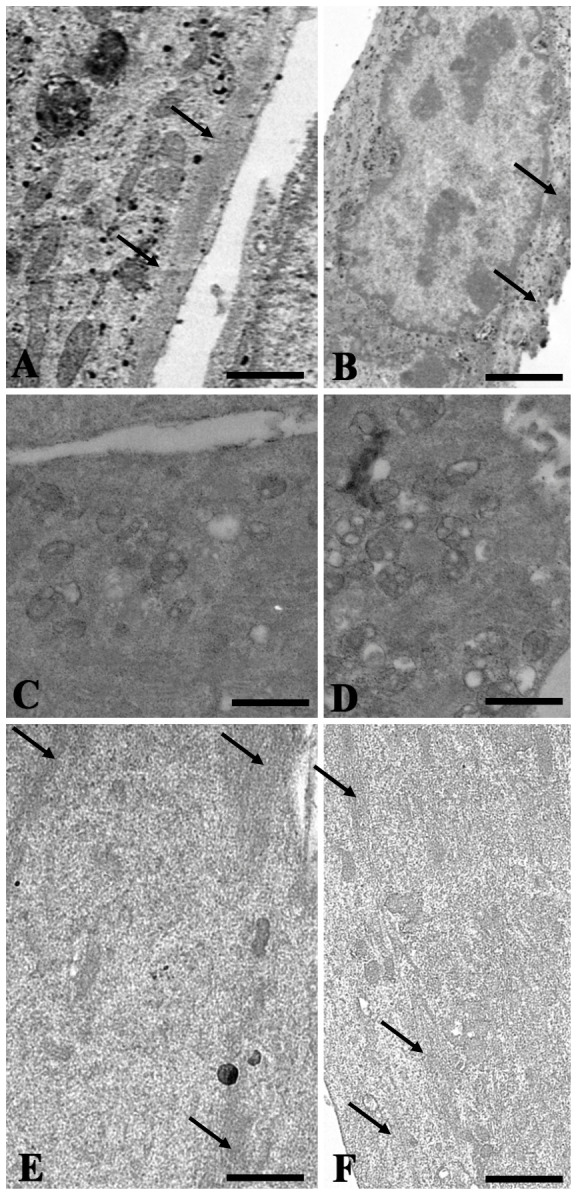
TEM of myofibrils (→) in myotube in control (**A**,**B**) exposed to inflammatory stress IL-1B (**C**) and LPS (**D**) and also in co-treatment with M-HA; µg/mL (**E**,**F**). (**A**–**F**) Bar = 1 micron.

Additionally, transmission electron microscopy provided additional evidence of differentiation activation (Figure 10). Both control (A) and M-HA (B) pre-treated cells displayed immature myofibrils that had not yet assembled into well-defined sarcomeres, indicating that differentiation was still in progress. Conversely, no myofibrillar structures were detected in cells treated with IL-1B (C) and LPS (D) pro-inflammatory agents, supporting the notion that inflammatory stimulation interferes with the differentiation process. Co- treatment with M-HA shows myofibrils similar to the control ones.

## 4. Discussion

The present study aimed to investigate whether in cell models of muscle damage and inflammation, M-HA, a multi-molecular-weight hyaluronic acid formulation, could ameliorate myoblast repair and preserve their myogenic capacity.

To this end, preliminary experiments were performed to determine whether M-HA affected cell viability toward the selected muscle-cell model, i.e., proliferating or differentiating C2C12 myoblasts. M-HA of up to concentrations five times higher than that used throughout the study (1.0 mg/mL) was neither toxic nor did it affect the rate of cell growth. In other words, our data indicate that M-HA complies with the basic requirements for its potential use in conditions requiring, in primis, the integrity and survival of the cells actively engaged in tissue repair [28]. These results align well with data from the literature, highlighting the general biocompatibility of HA for medical applications across different cell types [29], including muscle myoblasts [28]. In support of this view, we also observed a similar cytocompatibility profile in THP-1 monocytes, a cell model relevant to inflammatory processes, suggesting that the safety of M-HA may extend beyond the myogenic context.

A central aspect of our work concerns the ultrastructural observation combined with functional and molecular analyses, which together provide a comprehensive view of how M-HA modulates the response of muscle cells to inflammatory stress.

In line with previous descriptions of stress-related muscle damage [25], TEM examination of C2C12 myotubes exposed to LPS or IL-1β revealed typical signs of cellular distress, such as swollen mitochondria with poorly defined cristae, cytoplasmic rarefaction, and an increased number of autophagic vacuoles. In contrast, in the presence of M-HA LPS or IL-1β-challenged cells, the above ultrastructural alterations were not observed: mitochondria retained their double membrane and inner organization, and autophagic structures were markedly reduced.

Thus, the result of analyzing the mitochondrial size quantitatively supported our previous qualitative observation, showing an almost similar dimension in M-HA-treated cells and controls.

Interestingly, it has been reported that in human primary chondrocytes exposed to oxidative injury HA supplementation contributes to several activities, such as attenuating mitochondrial DNA damage, preserving ATP levels, and reducing apoptosis [30]. Though the cell type used herein is different, we also found HA-mediated protective effects against stressing conditions, suggesting that hyaluronan might have a similar cytoprotective mechanism that extends beyond a single cell type.

Taken together, these findings suggest that M-HA contributes to preserving mitochondrial homeostasis under stress, consistent with the protective role attributed to high-molecular-weight HA against insult-mediated injury and its interaction with CD44-dependent survival pathways [31,32].

In the present study, downstream CD44 signaling was not directly assessed. However, the protective effects of M-HA observed here, such as preservation of mitochondrial morphology, reduction of autophagic vacuoles, and recovery of the myogenic index, suggest the involvement of CD44-related survival pathways, including PI3K/AKT and ERK1/2, as reported in previous studies [33]. In addition, earlier reports indicate that HA–CD44 interactions contribute to the regulation of myogenic differentiation [34,35].

Notably, this structural protection seems to materialize into the functional behavior of the cells. In the scratch assay, M-HA enhanced wound closure by two-fold, not only under control conditions, but also in the presence of IL-1β and LPS, both of which are known to impair myoblast migration and regeneration through the activation of inflammatory signaling cascades, including NF-κB [36]. The improvement in repair capacity indicates that M-HA may help reorganize the pericellular matrix and support cytoskeletal dynamics, in agreement with the established role of HA–CD44 interactions in cell adhesion and motility [6,37].

The increase in myogenic index observed in M-HA-treated cultures indicates that the formulation does not merely support cell survival, but favors progression along the differentiation program. Consistently, treatment with M-HA was found to be associated with increased expression of MyoD, MRF4, and IGF-1, key regulators of myogenic commitment and differentiation [6,38]. These results align with those reported by Stellavato et al. [39], who found a significant enhancement of myogenic markers in muscle-derived cells treated with hybrid HA complexes. Our findings are also consistent with and extend those of Shi et al. (2023), who showed that HA-based hydrogel promotes myogenic differentiation of myoblasts and improves skeletal muscle regeneration in vivo in the rat tibialis anterior muscle defect model with reduced post-repair inflammatory factor expression [40].

From a translational perspective, it is also noteworthy that the same mixture of HA formulation investigated here has been tested in the context of tendon injuries—another common condition in sports medicine—without reported adverse effects [41]. Taken together, these converging observations support the concept that HA, whether delivered as a scaffold component or as a soluble multifractionated formulation, can help establish a microenvironment favorable to tissue repair, and that M-HA delivery within injured sites may represent a useful adjunct in regenerative strategies aimed at improving muscle recovery.

Additional considerations include the well-recognized decline of HA content in skeletal muscle, where it contributes to poor regeneration [42,43]. In this regard, M-HA formulation, which combines different molecular weights, may help to restore conditions that favor myoblast differentiation. Persisting inflammatory insults and inflammaging [44] usually suppress muscle repair capacity and impair muscle stem cell function [22,32]. The results indicate that, in our experimental setting, M-HA promoted the maintenance of a favourable transcriptional asset for myogenesis. At the same time and consistently, M-HA limited the expression of CCND1, counteracting the tendency of stressed cells to remain in a proliferative, poorly differentiated state. The preservation of MHC expression in M-HA-treated myotubes, despite IL-1β- and LPS-induced stress, confirms that the structural and contractile phenotype of differentiated cells is better maintained in the presence of M-HA. These effects are in line with previous reports showing that HA-based materials can support ECM organization and enhance muscle repair processes [45,46]. In particular, the use of blends or hybrid systems combining different HA molecular weight fractions has been proposed as a way to exploit both anti-inflammatory and pro-regenerative properties within the same product. Our findings are also consistent with those reported by Stellavato and colleagues, who demonstrated that HA-based gels protect primary rat muscle-derived cells from oxidative and atrophic stimuli, improve cell proliferation, and reduce the expression of atrophy [39]. Notwithstanding the differences in the experimental settings (cellular models and HA formulations), both Stellavato’s and our results support the idea that the multi-molecular weight hyaluronic acid formulation or hybrid HA preparations can modulate the response of muscle cells to stressful conditions and preserve their myogenic potential.

Further insight into the protective effects of M-HA is provided by the analysis of autophagy-related markers. Although autophagy is an important mechanism to control cellular homeostasis and mitochondrial maintenance under physiological conditions in skeletal muscles, its regulation can be disrupted under stress conditions [47]. In this study, for instance, autophagy-associated genes, including LC3B, beclin-1, Atg12, GABARAPL1, and cathepsin L, were upregulated in C2C12 cells treated with LPS or IL-1β. Therefore, this shows that the autophagic program is activated under stress conditions. In a similar study, skeletal muscles were exposed to inflammatory cytokines and endotoxins, and the autophagic gene expressions were increased. Thus, this long-lasting autophagic response led to impaired differentiation and muscle wasting [47,48]. Notably, the expression of autophagic-related genes was significantly reduced in cells treated with M-HA, while the basal expression of autophagy-associated genes was unaffected. Importantly, various myogenesis-related activities, such as mitochondrial function, cytoskeletal organization, and myotube maturation, can be affected by the impaired autophagy system.

These molecular findings confirm the ultrastructural observations obtained by TEM, where M-HA-treated cells displayed a marked reduction in autophagic vacuoles and preservation of mitochondrial morphology under inflammatory stress. The results obtained by transcriptional and ultrastructural approaches support the fact that M-HA contributes to maintaining a balanced autophagic flux, preventing the transition from an adaptive response to a potentially degenerative process. Under the biological point of view, this effect may be linked to the ability of M-HA to dampen inflammatory signaling and oxidative stress, both of which are potent upstream inducers of autophagy. This is achieved by stabilizing the pericellular matrix and engaging CD44-dependent survival pathways, preserving cellular homeostasis and supporting myogenic progression.

The ultrastructural analysis performed by transmission electron microscopy further supports that the differentiation program was activated in both control and M-HA treated cells. The presence of immature myofibrils that had not yet organized into mature sarcomeres is consistent with an ongoing differentiation process, reflecting an intermediate stage of myogenic maturation. In contrast, the absence of detectable myofibrillar structures in cells exposed to pro-inflammatory agents suggests that inflammatory stimuli impair the acquisition of the contractile apparatus, thereby interfering with normal myogenic differentiation. These findings are in agreement with the observed reduction in differentiation markers and support the hypothesis that inflammation negatively affects skeletal muscle cell maturation [49,50,51].

From a pharmacological point of view, it is important to emphasize that the M-HA selected for this study is a blend of multiple-sized HA fractions (see Section 2), ranging from small (<10 kDa) to medium (100–500 kDa) and high MW (≥1000 kDa), rather than a specific MW fraction. From the mechanistic point of view, this choice has been driven by the notion that the biological function of HA is reliant on its size [49], which influences the interaction with critical receptors regulating wound healing processes through several intracellular signaling pathways such as CD44, RHAMM, and TLR2 and 4 [52]. As these size-linked differential effects converge toward the promotion of tissue healing and repair [53] it is rational to focus on and point to the implementation for clinical purposes of multiple-sized HA formulations to treat muscular lesions. For this purpose, in an analogous manner to the established infiltrative techniques used for joints and tendons, HA preparations could be injected within the muscle intra- or peri-lesionally.

## 5. Conclusions

Our results provide compelling evidence that the combination of hyaluronic acid (HA) fractions with different molecular weights (M-HA) effectively protects C2C12 myoblasts and preserves their myogenic capacity under inflammatory stress. M-HA exerts a multifaceted protective action, safeguarding mitochondrial ultrastructure, sustaining myosin heavy chain (MHC) expression, and limiting excessive autophagic activation. These coordinated effects contribute to the maintenance of cellular integrity and promote a more efficient myofiber regenerative response. Notably, the preservation of both structural and functional hallmarks of myogenesis underscores the therapeutic potential of glycomics-based approaches for skeletal muscle repair. Although further in vivo investigations are needed to clarify the systemic mechanisms and long-term regenerative outcomes associated with M-HA treatment, the present findings establish a strong mechanistic framework supporting its biological activity. Collectively, these data position M-HA as a highly promising biomaterial for regenerative medicine applications, particularly in pathological settings characterized by persistent inflammation, oxidative stress, and impaired muscle healing.

## Figures and Tables

**Table 1:** Primer sequences for real-time PCR.

Primer Name	Sequence	
Myo-D	Forward Reverse	TTCTTCACCACTCCTCTGAC GCCGTGAGAGTCGTCTTAT
*IGF-1*	Forward Reverse	GCTATGGCTCCAGCATTCG TCCGGAAGCAACACTCATCC
*Mrf4*	Forward Reverse	GTGGCCAAGTGTTTCGGAT AAAGGCGCTGAAGACTGC
*CCND1*	Forward Reverse	CTTCCTCTCCAAAATGCCAG TGGAGGGTGGGTTGGAAAT
*LC3B*	Forward Reverse	CACTGCTCTGTCTTGTGTAGGTTG TCGTTGTGCCTTTATTAGTGCATC
*Beclin*	Forward Reverse	TGAATGAGGATGACACTCAGCA CACCTGGTTCTCCACACTCTTG
*Atg-12*	Forward Reverse	TCCGTGCCATCACATACACA TAAGACTGCTGTGGGGCTGA
*Gabarapl1*	Forward Reverse	CATCGTGGAGAAGGCTCCTA ATACAGCTGGCCCATGGTAG
*Cathepsin L*	Forward Reverse	GTGGACTGTTCTCACGCTCAAC TCCGTCCTTCGCTTCATAGG
*S16*	Forward Reverse	TGA AGG GTG GTG GAC ATG TG AAT AAG CTA CCA GGG CCT TTG A
*GAPDH*	Forward Reverse	GGCAAATTCAACGGCACAGT ACTCCACGACATACTCAGC

Note: CCND1, cyclin D1; Myo-D, myogenic differentiation 1; Mrf4, myogenic factor 4; IGF-1, insulin-like growth factor-1 isoform; LC3B, microtubule-associated protein 1A/1B-light chain 3B; Beclin; Atg-12, autophagy-related 12; Gabarapl1, GABA type A receptor-associated protein-like 1; Cathepsin L; S16, ribosomal protein S16; GAPDH, glyceraldehyde-3-phosphate dehydrogenase.

## Data Availability

Data are contained within the article and Supplementary Materials.

## Supplementary Materials

The following supporting information can be downloaded at: https://www.mdpi.com/article/10.3390/biom16060913/s1, Figure S1: Viability of THP-1 monocyte cell line treated with 1 or 5 mg/mL concentrations of M-HA for 24 and 48 h, as assessed by Trypan Blue, Figure S2: original image of Figure 9.

## Abbreviations

ATG12	Autophagy-related 12
BECN1	Beclin 1
CCND1	Cyclin D1
CTSL	Cathepsin L
DM	Differentiating medium
ECM	Extracellular matrix
GABARAPL1	GABA type A receptor-associated protein-like 1
GAPDH	Glyceraldehyde-3-phosphate dehydrogenase
HA	Hyaluronic acid
HAS2	Hyaluronan synthase 2
HMW-HA	High-molecular-weight HA
IGF-1	Insulin-like growth factor-1
IL-1β	Interleukin 1 beta
JMJD3	Histone demethylase Jumonji domain-containing 3
LC3B	Microtubule-associated protein 1A/1B-light chain
LPS	Lipopolysaccharide
LMW-HA	Low-molecular-weight HA
M-HA	Mixture of different molecular weight fractions of HA
MHC	Myosin heavy chain
MyoD	Myogenic differentiation
MRF4	Myogenic regulatory factor 4
MRFs	Myogenic regulatory factors
MuSCs	Muscle stem cells
TEM	Transmission electron microscopy
TGF-β	Transforming growth factor-β
TNF-α	Tumor necrosis factor-β
