# Supplementary material for: Revitalizing Muscle Repair: Hyaluronan Preserves Mitochondrial Architecture and Promotes Myogenesis Under Pro-Inflammatory Conditions
Source: Biomolecules. 2026 Jun 19;16(6):913. doi: 10.3390/biom16060913 (PMC13297520; doi:10.3390/biom16060913)
Supplement: Supplementary file 1 [file biomolecules-16-00913-s001.zip › biomolecules-4274466-Figure S1.pdf]

SUPPLEMENTAL MATERIAL

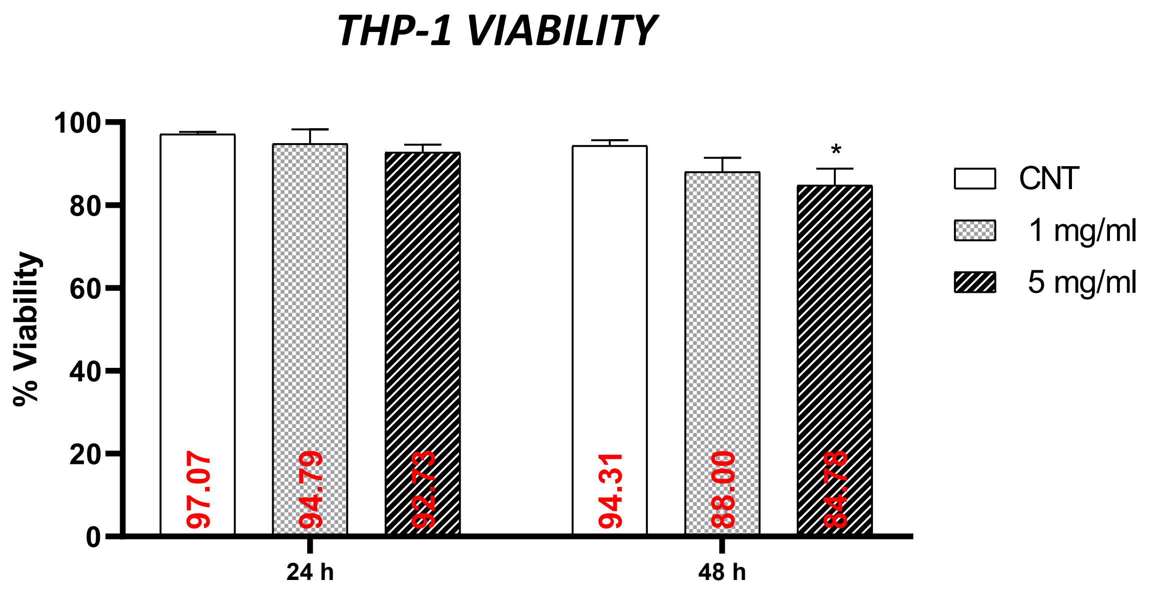

Figure S1. Viability of THP-1 monocyte cell line treated with 1 or 5 mg/ml concentrations of M-HA for 24 and 48 h, as assessed by Trypan Blue. Data are expressed as mean ± SD from n = 5 independent experiments, each performed in triplicate. Statistical analysis was carried out by two-way ANOVA - Sidak's multiple comparisons test; *p < 0.05, CNT *vs* M-HA.
