# Supplementary figures and images for: Revitalizing Muscle Repair: Hyaluronan Preserves Mitochondrial Architecture and Promotes Myogenesis Under Pro-Inflammatory Conditions
Source: Biomolecules. 2026 Jun 19;16(6):913. doi: 10.3390/biom16060913 (PMC13297520; doi:10.3390/biom16060913)

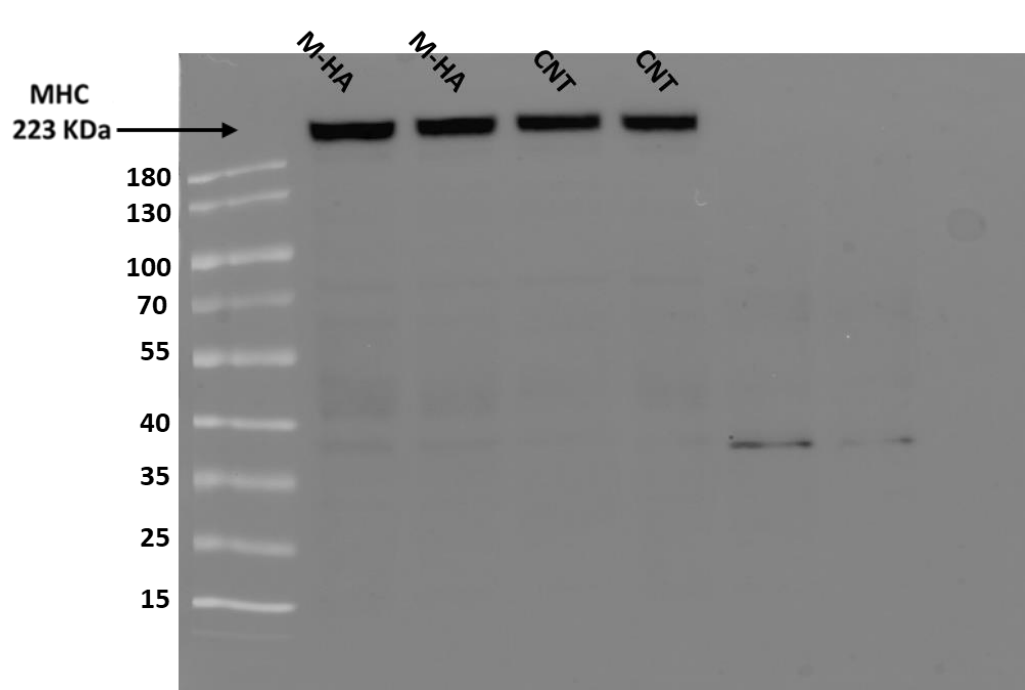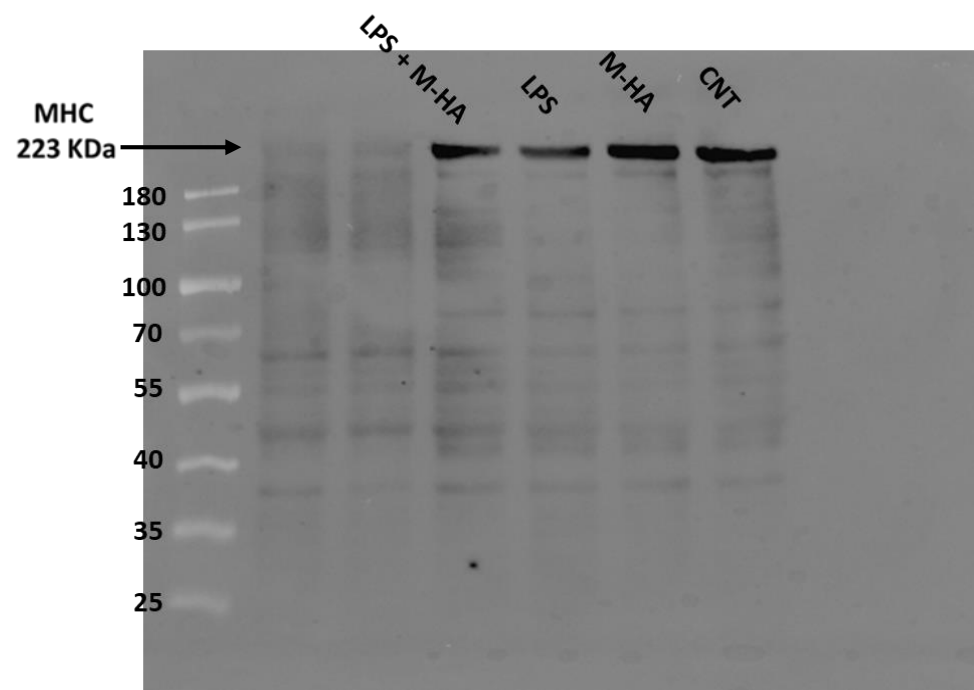

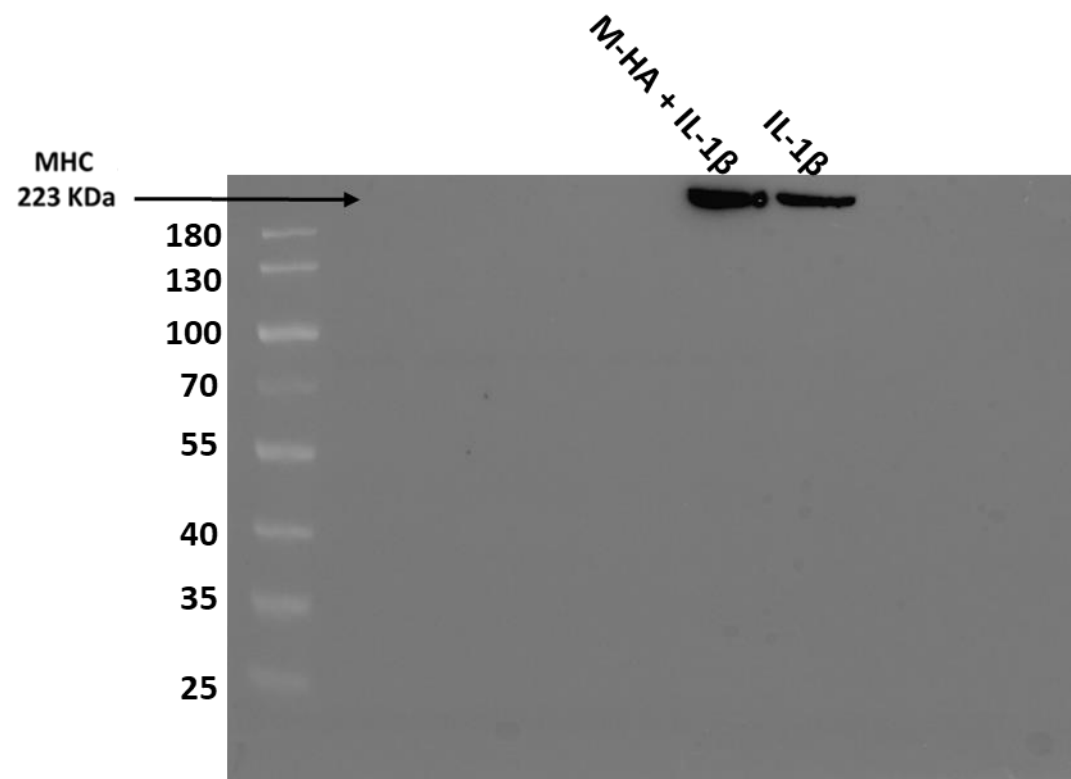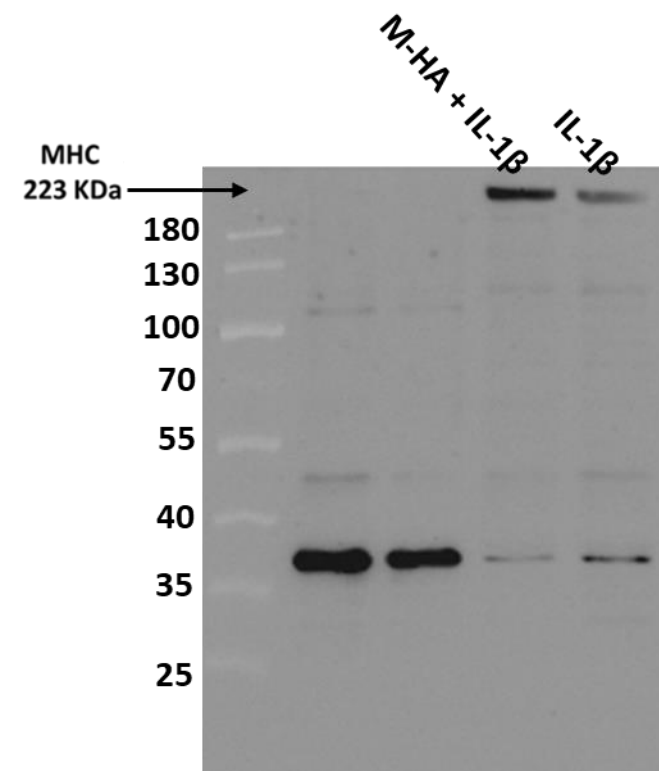



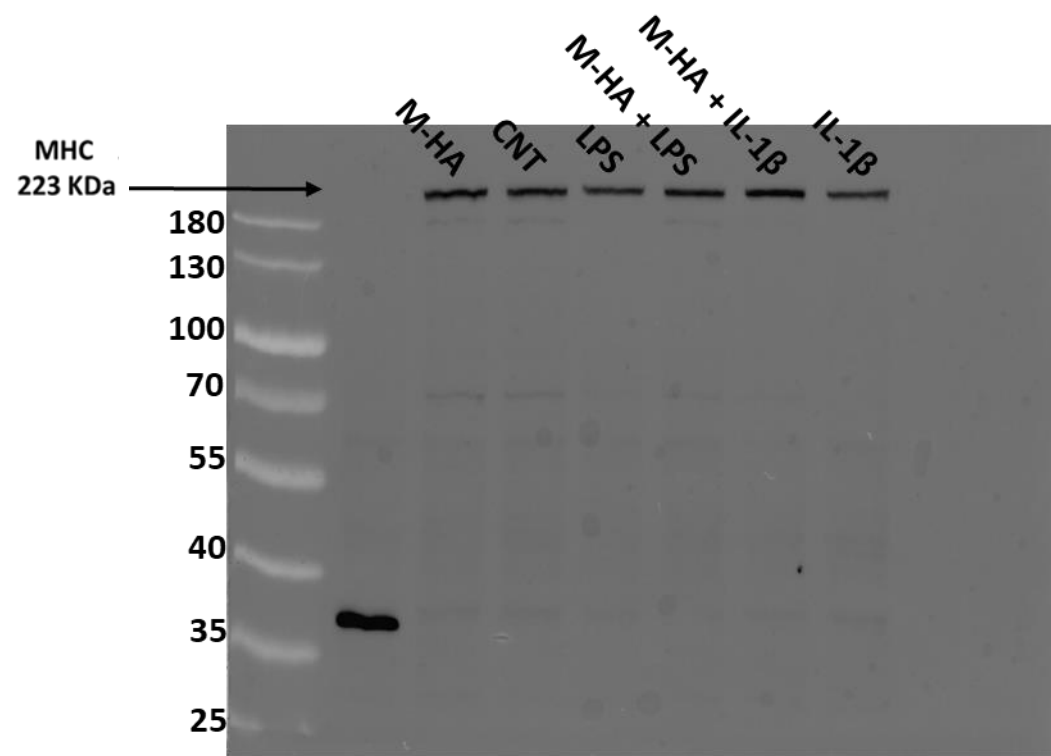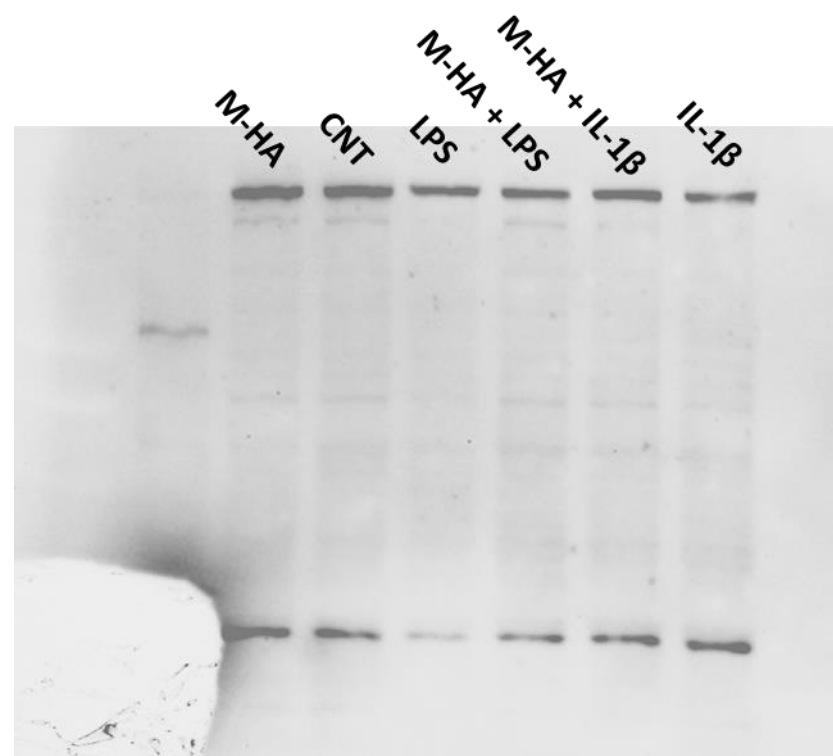

Supplement: Supplementary file 1 [file biomolecules-16-00913-s001.zip › biomolecules-4274466-Figure S2.pdf]
